# Validation of the generic medical interview satisfaction scale: the G-MISS questionnaire

**DOI:** 10.1186/s12955-017-0608-x

**Published:** 2017-02-14

**Authors:** Axel Maurice-Szamburski, Pierre Michel, Anderson Loundou, Pascal Auquier, For the G-MISS Study Investigators

**Affiliations:** 1 Laboratoire Universitaire EA 3279, Santé Publique et Maladies Chroniques, 27 boulevard Jean Moulin, Marseille, 13005 France; 2 Unité d’aide méthodologique, Direction de la Recherche Clinique, AP-HM, Marseille, France

**Keywords:** Patient experience, Satisfaction, General practice, Medical specialties, Surgical specialties, Communication, Relief, Compliance

## Abstract

**Background:**

Patients have about seven medical consultations a year. Despite the importance of medical interviews in the healthcare process, there is no generic instrument to assess patients’ experiences in general practices, medical specialties, and surgical specialties. The main objective was to validate a questionnaire assessing patients’ experiences with medical consultations in various practices.

**Method:**

The G-MISS study was a prospective multi-center trial that enrolled patients from May to July 2016. A total of 2055 patients were included from general practices, medical specialties, and surgical specialties. Patients filled out a questionnaire assessing various aspects of their experience and satisfaction within 1 week after their medical interview. The validation process relied on item response theory. Internal validity was examined using exploratory factorial analysis. The statistical model used the root mean square error of approximation, confirmatory fit index, and standard root mean square residual as fit indices. Scalability and reliability were assessed with the Rasch model and Cronbach’s alpha coefficients, respectively. Scale properties across the three subgroups were explored with differential item functioning.

**Results:**

The G-MISS final questionnaire contained 16 items, structured in three dimensions of patients’ experiences: “Relief”, “Communication”, and “Compliance”. A global index of patients’ experiences was computed as the mean of the dimension scores. All fit indices from the statistical model were satisfactory (RMSEA = 0.03, CFI = 0.98, SRMR = 0.06). The overall scalability had a good fit to the Rasch model. Each dimension was reliable, with Cronbach’s alpha ranging from 0.73 to 0.86. Differential item functioning across the three consultation settings was negligible. Patients undergoing medical or surgical specialties reported higher scores in the “Relief” dimension compared with general practice (83.0 ± 11.6 or 82.4 ± 11.6 vs. 73.2 ± 16.7; *P* < .001). A consultation shorter than 5 min correlated with low patient satisfaction in “Relief” and “Communication” and in the global index, *P* < .001.

**Conclusions:**

The G-MISS questionnaire is a valid and reliable questionnaire for assessing patients’ experiences after consultations with general practitioners, medical specialists, and surgical specialists. The multidimensional structure relies on item response theory and assesses different aspects of patients’ experiences that could be useful in clinical practice and research settings.

## Background

A patient has about seven doctors’ consultations per year [1]. Physicians are getting more involved in the quality assessment of daily practice [2], but few questionnaires have been validated to assess patients’ experiences with medical interviews [3, 4]. Patient-reported outcomes are considered valuable measures of healthcare and a step in the development of patient-centered care [5]. The assessment of patients’ experiences could enhance comparisons of strategies about physicians’ communication [6], treatment [7], or accountable care [8]. Available questionnaires in the field are not psychometrically sound [9], rely on expert generated items [3], and focus on specific physicians’ specialties [10] or specific patient courses [11, 12]. The Medical Interview Satisfaction Scale (MISS) was developed to assess patients’ experiences with interviews in primary care [4]. The authors used a rigorous method for item generation with patient interviews, but the factorial structure relied on the classical test theory (CTT) used at the time of questionnaire development [13]. Despite its predicted use in general practice, the original questionnaire tended to be a reference for the evaluation of patient-centered consultations [14–16]. Some questions were raised about the internal validity and acceptability of its 29 item form [17], and other authors suggested the factorial structure may differ across populations, stressing the need for a new validation process [18].

The main objective of the Generic Medical Interview Satisfaction Scale (G-MISS) study was to validate a generic version of the MISS questionnaire in general practice, medical specialties, and surgical specialties. The secondary objectives were to reduce the number of items and to explore the determinants of experience and satisfaction across patient groups, medical conditions, and consultation settings. The null hypothesis, defined as the lack of difference with the original questionnaire structure, was ruled out using Item Response Theory (IRT) with exploratory factorial analysis to assess patients’ experiences and satisfaction.

## Methods

### Patients

The protocol and statistical plan were approved by the Cerar ethical committee, Paris, France, ref. IRB 00010254-2016-023. The requirements of the Declaration of Tokyo were respected, and there was no interference in the physician-patient relationship.

All physicians, registered on the online health insurance server in the city of Marseille, France, were invited to participate in the study. Two thousand seventy-two physicians from various medical specialties were asked to enroll patients between May 2016 and July 2016. All consecutive adult patients undergoing medical consultations and able to complete a self-reported questionnaire were eligible. Non-inclusion criteria were the inability to fill an electronic form, cognitive impairment, and hospitalized patients.

### Protocol and data collection

All patients received written information at the time of online registration. Patient consent was obtained by electronic signature and stored in the server. Various specialties were represented including general practice, anesthesia, cardiology, dermatology, gynecology, gastroenterology, neurology, pulmonology, rheumatology, and the following surgeries: neurosurgery, cardiac, thoracic, maxillofacial, ear-nose-throat (ENT), orthopedic, plastic, urologic, vascular, visceral, and ophthalmologic. Physicians who actively participated in the study recruited the patients by giving them a single connection voucher.

Patients were asked to fill out an electronic form about their individual experience within 1 week after their consultation with the physician. There was no accompaniment of the patient in the formulation of the answers.

In accordance with the principle of the self-reported questionnaire, patients have to complete the form on their own, so that their answers reflected only their feelings about consultation. An introductory sentence mentioned that there was no right or wrong answer and that the questionnaire was anonymous and their responses confidential.

The questionnaire relied upon the original MISS-29, which contained 29 items structured in four dimensions named after their content: “Distress Relief” (11 items), “Communication comfort” (4 items), “Rapport” (10 items), and “Compliance intent” (4 items). The 29 items of the Medical Interview Satisfaction Scale were generated after interviews with patients, according to guidelines about item generation (see Appendix) [13]. Demographics were gathered along with sociocultural and medical condition data.

All personal information was anonymized before being sent to the server, according to the recommendations of the French national commission on information technology and human rights [19].

### Questionnaire reduction and validation

The questionnaire consisted of items structured into dimensions exploring the various aspects of patients’ experiences and satisfaction with medical consultations across three subgroups of physicians in general practice, medical specialties, and surgical specialties.

#### Item selection

We used the MISS-29 as an item bank to build the new questionnaire. A forward-backward translation was performed. A native English translator produced the first draft according to the original items. A bilingual expert back translated items to perform the cross-validation of the French version.

Items were considered for deletion if they loaded on two or more factors, or had a correlation of less than 0.40 with their own dimension according to the exploratory factorial analysis. Item deletion also relied on other standard criteria including inter-item correlation (lower than 0.40, or higher than 0.80), floor and ceiling effects (respectively higher than 15 and 40%) or low response rate (higher than 20% missing data). After the questionnaire’s multidimensional structure was identified, any item that, when deleted, would lead to a 0.02 increase in Cronbach’s alpha coefficient was removed.

#### Internal validity

An exploratory principal component factor analysis with varimax rotation [20] was performed along with inter-item, item-dimension, and inter-dimension correlations (Pearson r), to identify the questionnaire’s multidimensional structure. Each item was correlated with its own dimension and with the others.

If an item correlated higher (>0.4 after overlap correction) with its attendant dimension than with the others, the item internal consistency was supported, confirming its discriminant validity [21]. The internal consistency reliability of each potential dimension was assessed by Cronbach’s alpha coefficient with a threshold of 0.7 expected [22]. We used the polytomous Rasch model from IRT to explore the unidimensionality of the scale. This model assessed the ability of items to measure a “trait” or dimension of the scale. The Partial Credit Model, using threshold and discrimination parameters, was applied as an extension of the Rasch model [23, 24]. Each dimension’s scalability was explored by the pattern of item goodness-of-fit statistics (ranging from 0.5 to 1.5), ensuring that items belonging to the same dimension measured the same trait or concept [25].

#### Differential item functioning

We explored the differential item functioning (DIF) to assess the questionnaire properties across three settings of consultation, i.e. general practice, medical specialties, and surgical specialties. The DIF analysis sought to determine whether items and dimensions varied in their performance to assess patient satisfaction in these subgroups.

An increase in DIF would mean that the evaluated item functioned differently in the subgroup. The uniform DIF was calculated to determine the probability of giving a specific answer at a given level of satisfaction across physicians’ specialties.

The DIF was detected and the magnitude of the effect was quantified using the Crane and Larson model [26]. A significant DIF reports an increase in the explained variance of a given item when the subgroup’s variable is included, i.e. physicians’ specialties.

In case of statistical significance, Zumbo’s DIF classification was used to assess the DIF magnitude by computing delta R2. The DIF magnitude was considered negligible if delta R2 was <0.13, moderate if between 0.13 and 0.25, and large if >0.25.

#### External validity and acceptability

The original version of the MISS questionnaire reported correlation with occupational level [4]. Accordingly, we explored the external validity of the G-MISS across various groups of employment type. The G-MISS discriminant validity was further explored by comparisons between dimension scores and demographics, sociocultural levels, or medical conditions of patients using analysis of variance, Mann-Whitney *U*-test, and Pearson’s correlation. The online form of the questionnaire allowed the patient to skip any item without responding. The rate of missing data was assessed as an objective measure of acceptability [20].

Records showing fewer than 80% response rates were excluded from the validation analysis to ensure the quality of data.

#### Scoring

Items were answered using a five-point Likert scale, defined from 1 to 5 as “strongly disagree,” “disagree,” “neutral,” “agree,” and “strongly agree.” A dimension score was obtained by computing the mean of item scores for the dimension.

If less than one half of the items of a given dimension were missing, the mean of the non-missing items was substituted for scoring the dimension. Each dimension score was linearly transformed into a 0–100 scale with 0 indicating the worst level of satisfaction and 100 the best.

The global index was calculated as the mean of dimension scores. Dimensions were non-weighted, each was equal to the others for the computing the mean global index. Negatively phrased items were reversed when scored, so that higher scored items represented higher satisfaction (items 1, 5, 11, 15, 16, 18, 22, 24, 28, and 29) (see Appendix).

## Results

A total of 2055 patients were included in the study between May 2016 and July 2016 (see Fig. 2 Flow diagram). The baseline characteristics of the patients and types of consultations appear in Table 1. The psychometric validation resulted in a final version comprising 16 items structured into three dimensions, depending on their content: Relief (eight items), Communication (six items), Compliance (two items), Table 2. This short form explained 54.5% of the total variance.

**Table 1:** Patient’s characteristics and type of consultations (n = 1822)

Characteristics	N (%)
Gender	
Female	922 (49)
Male	900 (51)
Age	
18–23 years	419 (23)
24–33 years	476 (26)
34–47 years	449 (25)
48–75 years	478 (26)
Type of consultation	
General practice	505 (28)
Medical Specialties	615 (34)
Anesthesia	150 (8)
Cardiology	137 (8)
Dermatology	77 (4)
Gastroenterology	80 (4)
Neurology	34 (2)
Pulmonology	74 (4)
Rheumatology	63 (4)
Surgical Specialties	702 (38)
Cardiac and thoracic	40 (2)
ENT	43 (2)
Gynecology	133 (7)
Maxillofacial	35 (2)
Neurosurgery	28 (2)
Ophthalmology	80 (4)
Orthopedic	132 (7)
Plastic	86 (4)
Urologic	44 (2)
Vascular	35 (2)
Visceral	46 (2)
BMI	
Underweight	84 (5)
Normal	1299 (71)
Overweight	382 (21)
Obese	57 (3)
Tobacco	
Yes	677 (37)
No	1145 (63)
Consultation duration	
< = 5 min	223 (12)
10 min	477 (26)
15 min	481 (26)
20 min	348 (19)
25 min	205 (11)
> = 30 min	88 (5)
Emergency	
Yes	451 (25)
No	1371 (75)
Number of consultation in the last 6 months	
None	283 (16)
< 5	949 (52)
5–10	438 (24)
> 10	152 (8)
Hospitalization in the last 6 months	
Yes	378 (21)
No	1444 (79)
Long course treatment	
Yes	598 (33)
No	1224 (67)
Educational level	
Primary	24 (1)
Secondary	303 (17)
Bachelor	775 (43)
Master or above	720 (39)
Employment	
Without	410 (22)
Technician	254 (14)
Agent	254 (14)
Senior	265 (15)
Licensed professional	139 (8)
Retired	152 (8)
Other	347 (19)

*BMI* body mass index, *ENT* ear nose and throat

**Table 2:** Principal Component Analysis (Varimax rotation) and DIF of the G-MISS

Item n°	Relief	Communication	Compliance	DIF	Delta R2
Q3	**0.840**			0,43	
Q4	**0.803**			0,93	
Q2	**0.767**			0,31	
Q6	**0.669**			**0,00**	0,01
Q25	**0.668**			0,71	
Q1	**0.639**			0,13	
Q24	**0.600**			0,50	
Q19	**0.560**			**0,00**	0,01
Q13		**0.697**		0,12	
Q12		**0.679**		0,11	
Q11		**0.669**		**0,02**	0,00
Q15		**0.627**		**0,03**	0,00
Q9		**0.607**		0,07	
Q8		**0.561**		0,35	
Q28			**0.915**	**0,00**	0,01
Q26			**0.902**	**0,01**	0,00

For clarity, factor loadings below 0.3 are not reported in the table. For each column bold numbers are the factor loadings of the items participating in the computation of the corresponding dimension. DIF are expressed as Chi2-*p*-value

*G–MISS* Generic–Medical Interview Satisfaction Scale

### Sample characteristics

Patients enrolled in the validation process were consulted at various medical and surgical specialties *n* = 1822 (Table 1). The mean patient age was 37.1 ± 14.7 years and 451 consultations were made in an emergency setting (25%). Five hundred ninety-eight patients (33%) reported a long course treatment and 378 had been hospitalized in the last 6 months (21%). This was the first consultation in 6 months for 283 patients (16%). Four hundred and ten patients were unemployed at the time of the consultation (22%) (Table 1).

### Internal validity

**Fig. 1:**
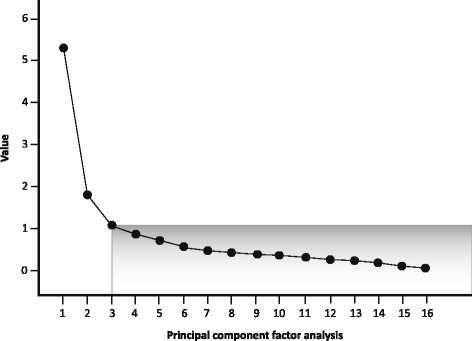
Number of component scree plot of the 16-items G-MISS

The G-MISS final version contained 16 items structured in a three-factor questionnaire determined by exploratory factor analysis (Fig. 1). Thirteen items were suppressed according to their load factor and retained suppression criteria. The three dimensions were named after their item content, according to the original version of the MISS-29 questionnaire: “Relief” (eight items), “Communication” (six items), and “Compliance” (two items). The “Rapport” dimension from the original MISS-29 was removed and its remaining items were merged into the “Communication” dimension according to the exploratory factorial analysis. All the fit indices from the statistical model were satisfactory (RMSEA = 0.03, CFI = 0.98, SRMR = 0.06). The overall scalability was good, with items showing a good fit to the Rasch model in each dimension. Item internal consistency (IIC) was satisfactory for all dimensions; each item achieved the 0.40 standard threshold for IIC (ranging from 0.45 to 0.77, Table 3). The correlation of each item with its contributive dimension was higher than those with other dimensions (item discriminant validity, IDV). Cronbach’s alpha coefficients ranged from 0.73 to 0.86, indicating satisfactory reliability for each dimension.

**Table 3:** Internal validity

Dimensions	Mean ± SD	IIC	IDV	Alpha	INFIT
Global population (*n* = 1822)					
Relief (8)	80.0 ± 13.8	0.50–0.77	0.11–0.37	0.86	0.59–1.18
Communication (6)	76.3 ± 13.4	0.42–0.61	0.14–0.39	0.73	0.70–0.92
Compliance (2)	76.5 ± 19.5	0.72–0.72	0.18–0.26	0.84	0.50–0.51
Index (16)	78.2 ± 11.1	NA	NA	0.85	NA
General practice (*n* = 505)					
Relief (8)	73.2 ± 16.7	0.58–0.61	0.18–0.36	0.89	0.56–1.16
Communication (6)	74.6 ± 13.3	0.42–0.61	0.14–0.36	0.74	0.66–0.98
Compliance (2)	76.5 ± 16.9	0.64–0.64	0.22–0.31	0.78	0.48–0.48
Index (16)	74.1 ± 12.1	NA	NA	0.86	NA
Medical specialties (*n* = 615)					
Relief (8)	82.1 ± 12.4	0.38–0.74	0.06–0.37	0.82	0.57–1.21
Communication (6)	77.6 ± 13.2	0.40–0.53	0.05–0.43	0.71	0.71–0.95
Compliance (2)	76.0 ± 21.2	0.75–0.75	0.20–0.23	0.86	0.52–0.53
Index (16)	79.7 ± 10.4	NA	NA	0.82	NA
Surgical specialties (*n* = 702)					
Relief (8)	83.1 ± 10.9	0.41–0.66	0.10–0.39	0.81	0.66–1.13
Communication (6)	76.4 ± 13.6	0.47–0.58	0.15–0.44	0.75	0.72–0.91
Compliance (2)	76.5 ± 19.7	0.75–0.75	0.19–0.28	0.86	0.50–0.51
Index (16)	78.2 ± 11.1	NA	NA	0.84	NA

Data are presented as mean ± SD. Dimension scores range from 0 (lowest satisfaction) to 100 (highest satisfaction). IDV: correlation between items scores of a given dimension with the other dimension scores. Numbers are lowest–highest Pearson correlation coefficient. IIC: correlation between items scores and their dimension score (corrected for overlap). Numbers are lowest–highest Pearson correlation coefficient. *IDV* item-discriminant validity, *IIC* item-internal consistency, *NA* not applicable, *INFIT* item goodness-of-fit

Floor effects ranged from 0.3 to 13.2%, and ceiling effects ranged from 18.2 to 36.9% (data not shown). Patients who finished the questionnaire responded to all items. The rate of missing values was low (see Applicability). According to the definition of DIF, six items (q8, q11, q19, q25, q26, q28) showed a statistically significant difference in their behavior according to the consultation specialty, but the magnitude of the DIF was negligible for each (Table 2).

### External validity

The educational level and type of employment correlated with the “Communication” dimension and with the global index of the G-MISS. The type of employment also correlated with the “Relief” dimension.

Patients consulted by medical and surgical specialties showed higher satisfaction scores in the “Relief” dimension than those that attended general practices (83.0 ± 11.6 and 82.4 ± 11.6 vs. 73.2 ± 16.7, respectively; *P* < .001), but the most relevant discrepancies were reported for the global index (80.5 ± 9.9, 79.2 ± 10.4 vs. 74.1 ± 12.1, respectively; *P* < .001, Table 4). There were not differences in the “Compliance” dimension between physician specialties. There was no difference in terms of age, sex, or body mass index (BMI). Smoker patients showed lower satisfaction scores in the “Communication” and “Compliance” dimensions, and in the global index. Consultations shorter than 5 min correlated highly with low patient satisfaction in the “Relief” and “Communication” dimensions, and in the global index, *P* < .001. The “Compliance” dimension was not correlated with the duration of consultations (Table 4). The emergency context correlated with the “Relief” dimension and global index. There was a linear association between the number of consultations in the last 6 months and the level of satisfaction in the “Relief” dimension, *P* = .001.

**Table 4:** Comparisons of G-MISS scores according to patient’s characteristics and type of consultations (*n* = 1822)

Characteristics	Relief	Communication	Compliance	Index
Gender				
Female	80.2 ± 13.5	75.8 ± 13.7	76.3 ± 19.8	78.0 ± 11.2
Male	79.9 ± 14.2	76.9 ± 13.1	76.3 ± 19.2	78.3 ± 10.9
*t-test*	NS	NS	NS	NS
Effect size	0.02	0.08	0.01	0.02
Age				
18–23 years	80.3 ± 15.2	76.6 ± 13.8	78.0 ± 18.5	78.6 ± 11.5
24–33 years	78.9 ± 13.9	76.2 ± 13.5	76.9 ± 19.2	77.7 ± 11.2
34–47 years	79.8 ± 13.4	75.5 ± 13.2	75.2 ± 19.6	77.6 ± 10.5
48–75 years	81.1 ± 12.9	77.0 ± 13.3	75.4 ± 20.4	78.8 ± 11.0
*ANOVA*	NS	NS	NS	NS
Effect size	0.01	0.01	0.01	0.01
Type of consultation				
General practice	73.2 ± 16.7	74.6 ± 13.3	76.5 ± 16.9	74.1 ± 12.1
Medical Specialties	83.0 ± 11.6	78.6 ± 12.5	76.3 ± 21.6	80.5 ± 9.9
Surgical Specialties	82.4 ± 11.6	75.9 ± 13.9	76.3 ± 19.5	79.2 ± 10.5
*ANOVA*	0.000	0.000	NS	0.000
Effect size	0.09	0.01	0.01	0.05
BMI				
Underweight	81.7 ± 12.8	74.6 ± 13.6	78.7 ± 19.4	78.6 ± 11.5
Normal	79.9 ± 14.2	76.5 ± 13.3	76.9 ± 18.6	78.2 ± 11.1
Overweight	79.9 ± 12.7	76.6 ± 13.0	74.5 ± 20.7	78.0 ± 10.1
Obese	81.4 ± 14.2	73.4 ± 18.6	73.5 ± 24.7	77.4 ± 14.6
*ANOVA*	NS	NS	NS	NS
Effect size	0.01	0.01	0.01	0.01
Tobacco				
Yes	79.5 ± 13.9	75.4 ± 13.9	73.7 ± 21.4	77.2 ± 11.5
No	80.3 ± 13.8	76.9 ± 13.1	77.9 ± 18.1	78.7 ± 10.8
*t-test*	NS	0.026	0.000	0.005
Effect size	0.06	0.11	0.21	0.13
Consultation duration				
< = 5 min	73.7 ± 17.1	73.4 ± 13.9	77.1 ± 17.9	74.0 ± 12.6
10 min	80.0 ± 14.0	78.0 ± 12.9	77.2 ± 18.9	78.9 ± 10.9
15 min	80.7 ± 13.0	76.6 ± 13.7	76.1 ± 20.0	78.6 ± 10.8
20 min	81.8 ± 12.8	76.5 ± 12.4	76.6 ± 19.3	79.2 ± 10.3
25 min	82.1 ± 10.8	75.2 ± 14.0	74.9 ± 20.1	78.6 ± 10.1
> = 30 min	80.7 ± 14.1	74.6 ± 14.8	73.5 ± 22.8	77.5 ± 12.2
*ANOVA*	0.000	0.001	0.503	0.000
Effect size	0.05	0.01	0.01	0.03
Emergency				
Yes	81.4 ± 13.9	77.4 ± 13.6	76.4 ± 20.7	79.3 ± 10.9
No	79.6 ± 13.8	76.0 ± 13.4	76.3 ± 19.1	77.8 ± 11.1
*t-test*	0.017	0.054	NS	0.016
Effect size	0.13	0.10	0.01	0.13
Number of consultation in the last 6 months				
None	77.2 ± 16.7	73.7 ± 15.2	74.2 ± 20.9	75.5 ± 12.5
< 5	80.0 ± 13.4	76.5 ± 13.0	77.3 ± 18.6	78.4 ± 10.9
5–10	81.4 ± 12.8	77.4 ± 12.9	76.5 ± 19.5	79.3 ± 10.3
> 10	81.5 ± 13.2	76.8 ± 13.8	74.0 ± 21.9	78.8 ± 10.6
*ANOVA*	0.001	0.004	0.046	0.000
Effect size	0.01	0.01	0.01	0.01
Hospitalization in the last 6 months				
Yes	81.8 ± 12.1	76.8 ± 13.0	74.6 ± 21.3	79.0 ± 10.2
No	79.6 ± 14.2	76.2 ± 13.5	76.8 ± 19.0	77.9 ± 11.3
*t-test*	0.005	NS	0.051	NS
Effect size	0.17	0.05	0.11	0.10
Long course treatment				
Yes	80.8 ± 13.5	76.9 ± 13.2	75.5 ± 21.2	78.7 ± 11.0
No	79.6 ± 14.0	76.0 ± 13.5	76.8 ± 18.6	77.9 ± 11.1
*t-test*	NS	NS	NS	NS
Effect size	0.08	0.07	0.06	0.07
Educational level				
Primary	78.1 ± 16.7	78.1 ± 14.4	78.8 ± 19.4	78.2 ± 12.4
Secondary	79.4 ± 14.3	74.5 ± 15.2	74.3 ± 21.5	76.9 ± 12.0
Bachelor	79.4 ± 13.5	75.8 ± 13.2	76.3 ± 19.1	77.7 ± 11.0
Master or above	81.0 ± 14.0	77.5 ± 12.7	77.1 ± 19.0	79.2 ± 10.6
*ANOVA*	NS	0.006	NS	0.009
Effect size	0.01	0.01	0.01	0.01
Employment				
Without	79.1 ± 14.3	75.3 ± 13.9	73.5 ± 20.8	77.0 ± 11.6
Technician	77.8 ± 15.5	74.5 ± 14.7	77.6 ± 18.1	76.5 ± 12.2
Agent	80.2 ± 12.3	76.4 ± 12.8	76.5 ± 19.9	78.3 ± 9.8
Senior	80.7 ± 13.6	78.3 ± 11.8	77.8 ± 19.0	79.4 ± 9.8
Licensed professional	80.8 ± 13.3	76.6 ± 12.3	76.8 ± 20.0	78.7 ± 10.2
Retired	81.3 ± 13.4	77.6 ± 13.4	77.6 ± 18.5	79.4 ± 11.2
Other	81.2 ± 13.5	76.6 ± 13.8	76.7 ± 19.0	78.9 ± 11.4
*ANOVA*	0.045	0.025	NS	0.008
Effect size	0.01	0.01	0.01	0.01

*BMI* body mass index, *ANOVA* Analysis of variance. *t-test* Student *t*-test

Hospitalization in the last 6 months was associated with a higher level of satisfaction in the “Relief” dimension (*P* = .005). A long course of treatment was not correlated with overall satisfaction scores on the G-MISS.

### Applicability

**Fig. 2:**
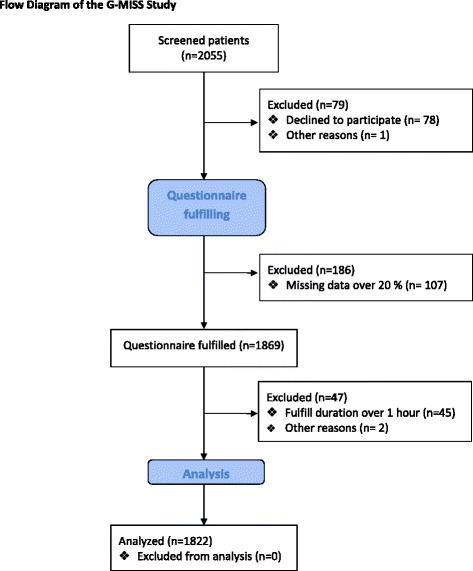
Flow Diagram of the G-MISS Study

Seventy-eight patients declined to participate after being screened (3.8%). One hundred and seven patients showed a rate of missing values >20% (5.2%). The percentage of missing values increased along with the progression of the questionnaire. Patients with a rate of missing values >20% did not respond to any items from number 15 (data not shown). Forty-five patients (2.2%) were excluded because they took more than 1 h to fill out the 29 item questionnaire (see Fig. 2 Flow diagram). The mean filing duration for the 16 items of the G-MISS was 6 min and 49 s, [±2 min, 41 s], vs. 12 min, 23 s [±5 min, 13 s] for the original set of 29 items, *P* < .001.

## Discussion

Patient-reported outcome evaluations have become mandatory [27, 28]. Most regulatory authorities in Organisation for Economic Co-operation and Development (OECD) have included such outcomes in their quality assessment framework. In the United States, the Primary Care Assessment Survey (PCAS) was initially developed to measure the quality of service in seven domains of primary care through 11 scales [29] and was further developed and tested to assess performance [30]. The Consumer Assessment of Healthcare Providers & Systems (CAHPS) is used in the hospital setting to discriminate between strategies in Medicare Accountable Care Organizations [8].

In Europe, the UK’s Quality of Outcomes Framework was introduced in 2004 [31]. The General Practice Assessment Questionnaire (GPAQ) was derived from the original PCAS [32–34] and its psychometrical properties were retrospectively tested [35]. The GPAQ is used by the UK department of Public Health and Primary Care as a survey for general practitioners’ revalidations and practices [36]. These tools are oriented toward quality of service and performance assessments, but there is no generic tools forecasted to the evaluation of patient experience itself in various practice. However, patient satisfaction could be misleading if evaluated with tools that are validated to assess quality of service or inherited from the consumer field [5]. Taking into account patient expectations should help physicians with patient interactions and shared decision-making [37, 38].

In our study, the null hypothesis was rejected according to the differences that emerged between the original MISS-29 structure and that of the G-MISS short form reported here. The MISS-29 was specifically developed to assess patients’ experiences and expectations about physician consultations. The item generation relied on patient interviews to assess the cognitive, affective, and behavioral dimensions of patient-physician interactions [4]. The original set of items demonstrated good wording and comprehension compared with the Consultation Satisfaction Questionnaire [3]. The 29 item form was criticized for its acceptability, with a mean filling time of 12 min. This encouraged some authors to develop a short form of the MISS-29, for use only in general practice [17].

The purpose of the present study was to develop a questionnaire suitable for most consultation settings, by using IRT to select items and structure them into dimensions of experience.

One particular advantage of IRT over CTT is its independence in regard to the population being tested. The G-MISS final version is a self-reported questionnaire of 16 items structured into three dimensions of patients’ experiences and satisfaction with doctors’ consultations. One strength of the questionnaire is its large validation sample of 1822 patients, who were consulted in various general practices as well as medical and surgical specialties. The process of selection deleted items with equivocal loading into the factorial structure. There was no item switch from a dimension to another. The items belonging to the original “Rapport” and “Communication comfort” dimensions were merged into a single dimension, “Communication”, after the exploratory factorial analysis. The G-MISS questionnaire reported high levels of internal validity across its three dimensions, confirming that patients’ experiences are a multidimensional concept. Accordingly, the reliability indices of the three dimensions were satisfactory.

No notable differential item functioning was reported, ensuring that the questionnaire is equally reliable in every setting of consultation it was forecasted for, i.e. general practice, medical specialties and surgical specialties. The three-dimensional structure assesses different aspects of patients’ experiences. The G-MISS dimensions were named after the original MISS-29 questionnaire [13].

The “Relief” dimension assesses the alleviation of illness-related stress. It has been reported that patients experiencing symptoms of chronic or acute conditions worry about the potential impact of their disease on their life [39]. By relieving stress, physicians could improve health status and well-being [40]. The “Communication” dimension assesses the communication comfort between the patient and doctor.

It has been previously reported that patients consider communication to be one of the most important physician skills [41], but even if technically sound, the physician’s communication may appear inappropriate to the patient [42]. A recent systematic review stressed the lack of tools to assess physician’s communication [43]. The “Compliance” dimension reports the patient’s intent to follow doctor’s recommendations. It is well known that medical consultation influences patient compliance; [44] for example, specific attention should be given to verbal interactions, especially in long-term treatments like blood pressure control programs [45].

The items from the “Compliance” dimension were below the usual range of acceptance for the Infit in the general practice population [25]. One could hypothesize that there is an overlap between compliance and communication or relief, because of an indirect connection between these dimensions. A systematic review pointed out the link between physician-patient communication and outcomes in primary care [43].

The level of explanation given by the physician has been correlated with patient compliance [45] and could have influenced the “Compliance” and “Relief” or “Communication” dimensions assessed by the G-MISS scale. In contrast, unadapted behavior from a physician demonstrating nervousness or anger has been associated with less compliance from the patient and could also influence several dimensions of the patient’s experience [46]. The level of Infit was satisfactory in the global population, medical specialties, and surgical specialties, for all dimensions.

The level of satisfaction in the “Relief” dimension was significantly lower in general practice than medical or surgical specialties.

Some patients may not feel sufficiently reassured by their general practitioner’s explanations and need a referral to a specialist [39]. Specialists could be more likely to use situation-specific reassurance strategies while standard communication skills tend to stay generic [47]. These “Relief” dimension findings translated into the results for the global index with specialists’ interviews reporting higher satisfaction scores. Lack of practitioner’s time is classically reported as a cause of dissatisfaction for patients [48].

Although the perceived consultation duration has been associated with better patient satisfaction, the actual length of consultation has produced controversial results [49, 50]. One could hypothesize that the use of non-validated questionnaires may have explained the lack of difference [51]. In our study, interviews that lasted less than 5 min were significantly correlated with lower satisfaction scores in the “Relief” and “Communication” dimensions and global index. The G-MISS correlated with patients’ type of employment which is concordant with the original questionnaire. The external validity of the G-MISS scale was further emphasized by correlations with the emergency context, the educational level and the number of consultations in the last 6 months.

Among patients with a rate of missing values >20%, the number of responded items dramatically fell from item 15 to 29. This reinforces the interest for a short-form to improve the applicability of an everyday practice questionnaire. The final version of the G-MISS questionnaire is 16 items long and took about 6 min to complete. Despite this short form, the G-MISS questionnaire still explained 54.5% of the total variance.

The level of education wasn’t correlated with the rate of missing answers, suggesting that the questionnaire is widely administrable to patients able to read and complete an online survey. Application abilities in various medical fields of primary care underline the interest for the questionnaire in a patient-centered care approach.

## Conclusion

The G-MISS questionnaire is a valid and reliable short-form questionnaire to assess patients’ experiences and satisfaction with physician consultations in general practices, medical specialties, and surgical specialties. The multidimensional structure relies on IRT and assesses different aspects of patients’ experiences that could be useful in clinical practice and research settings.

## Appendix

**Table 5:** MISS-29 original and translated questionnaire

Items	Original	French version
1^a^	The doctor gave me a poor explanation of my illness	Le médecin m’a donné peu d’explication concernant ma maladie
2	The doctor told me what my illness is	Le médecin m’a dit de quoi j’étais malade
3	After talking with the doctor, I know just how serious my illness is	Après avoir parlé au médecin, je connais exactement le degré de la gravité de ma maladie
4	The doctor told me all I wanted to know about my illness	Le médecin m’a dit tout ce que je voulais savoir à propos de ma maladie
5^a^	I am not really certain how to follow the doctor’s advice	Je ne sais pas comment suivre les conseils du médecin
6	After talking with the doctor, I have a good idea of how long it will be before I am well again	Après avoir parlé au médecin, j’ai une bonne idée du temps nécessaire avant que j’aille mieux
7	The doctor seemed interested in me as a person	Le médecin a semblé s’intétesser à moi en tant que personne
8	The doctor seemed warm and friendly to me	Le médecin m’a semblé chaleureux et amical
9	I felt this doctor did not treat me as an equal	J’ai senti que ce médecin ne m’a pas traité d’égal à égal
10	The doctor seemed to take my problems seriously	Le médecin a semblé prendre en compte mes problèmes sérieusement
11^a^	I felt embarrassed while talking with the doctor	Je me suis senti géné de parler avec le médecin
12	I felt free to talk to this doctor about private matters	Je me suis senti libre d’aborder ma vie privée avec le médecin
13	The doctor gave me a chance to say what was really on my mind	Le médecin m’a donné la possibilité de dire ce que je pensais vraiment
14	I really felt understood by my doctor	Je me suis senti réellement compris par le médecin
15^a^	The doctor did not allow me to say everything I had wanted about my problems	Le médecin ne m’a pas permit de dire tout ce que j’aurais voulu dire concernant mes problèmes
16^a^	The doctor did not really understand my reason for coming	Le médecin n’a pas vraiment compris les raison de ma visite
17	This is a doctor I would trust my life with	C'est un médecin auquel je confierais ma vie
18^a^	I would hesitate to recommend this doctor to my friends	J’hésiterais à recommender ce médecin à mes amis
19	The doctor seemed to know what he/she was doing	Le médecin semblait savoir ce qu’il/elle faisait
20	After talking with the doctor, I feel much better about my problems	Après avoir parlé au médecin, je me sens beaucoup mieux concernant mes problèmes
21	The doctor has relieved my worries about my illness	Le médecin a soulagé mes inquietudes concernant ma maladie
22^a^	Talking with my doctor has not at all helped my worries about my illness	Parler avec le médecin n’a pas soulagé mes inquietudes par rapport à ma maladie
23	The doctor has come up with a good plan for helping me	Le médecin a établi une bonne stratégie pour m’aider
24^a^	The doctor’s visit has not at all helped me	La consultation du médecin ne m’a pas aidé du tout
25	The doctor seemed to know just what to do for my problem	Le médecin semblait savoir exactement quoi faire pour mon problème
26	I expect that it will be easy for me to follow the doctor’s advice	Je pense que ce sera facile pour moi de suivre les conseils du médecin
27	I intend to follow the doctor’s instructions	J’ai l’intention de suivre les instructions du médecin
28^a^	It may be difficult for me to do exactly what the doctor told me to do	Ce pourrait être difficile pour moi de faire exactement ce que le médecin m’a dit de faire
29^a^	I am not sure the doctor’s treatment will be worth the trouble it will take	Je ne suis pas sûr que le traitement préscrit par le médecin en vaut la peine

^a^ Item reversed to be score, so that higher score represents higher satisfaction

## Abbreviations

BMI: Body mass index
CAHPS: Consumer Assessment of Healthcare Providers & Systems
CTT: Classical test theory
DIF: Differential Item Functionning
ENT: Ear Nose Throat
G-MISS: Generic Medical Interview Satisfaction Scale
GPAQ: General Practice Assessment Questionnaire
IDV: Item discriminant validity
IIC: Item internal consistency
IRT: Item Response Theory
MISS: Medical Interview Satisfaction Scale
OECD: Organisation for Economics Co-operation an Development
PCAS: Primary Care Assessment Survey
